# Supplementary figures and images for: Chemical Bonding in Three-Membered Ring Systems
Source: Molecules. 2025 Jan 30;30(3):612. doi: 10.3390/molecules30030612 (PMC11820764; doi:10.3390/molecules30030612)

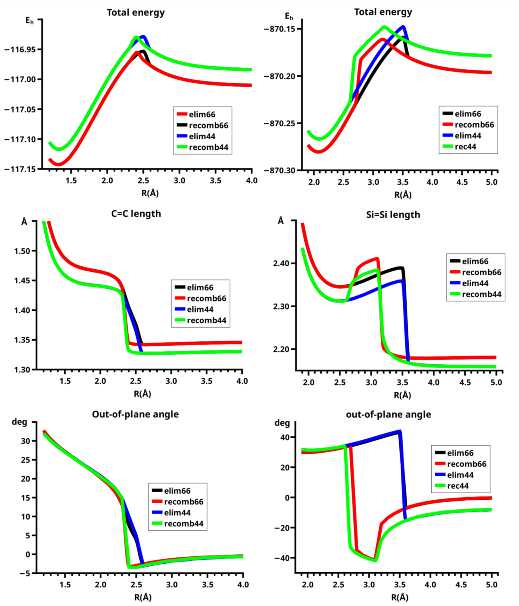

Supplement: Supplementary file 1 [file molecules-30-00612-s001.zip › Figure53.png]

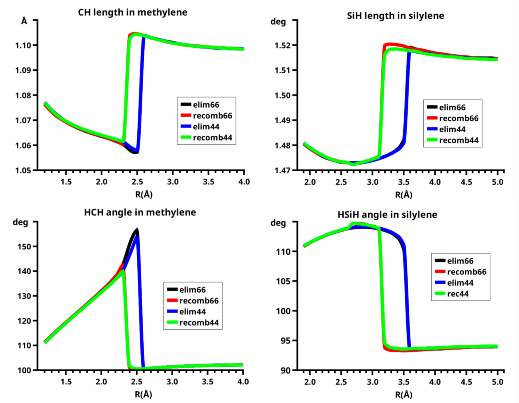

Supplement: Supplementary file 1 [file molecules-30-00612-s001.zip › Figure54.png]

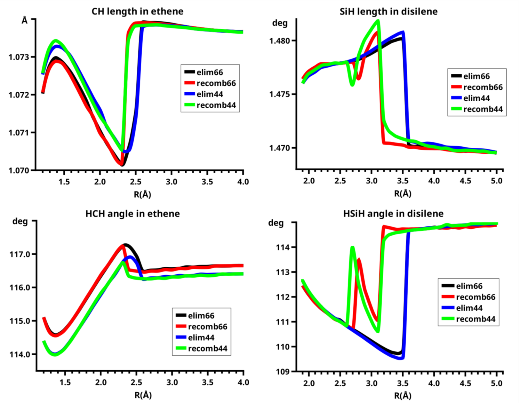

Supplement: Supplementary file 1 [file molecules-30-00612-s001.zip › Figure55.png]

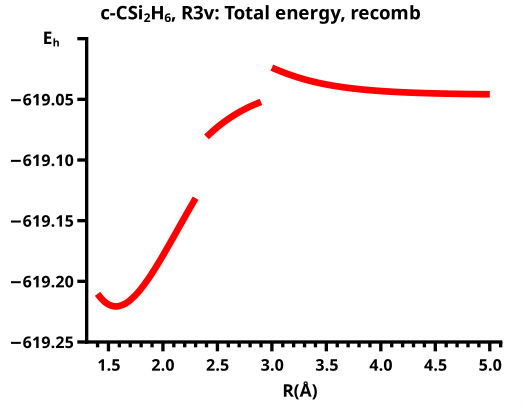

Supplement: Supplementary file 1 [file molecules-30-00612-s001.zip › Figure56.png]

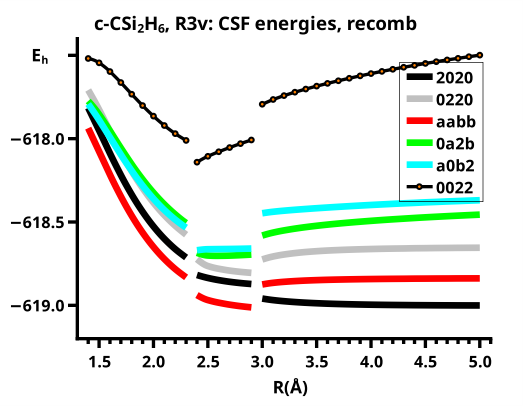

Supplement: Supplementary file 1 [file molecules-30-00612-s001.zip › Figure57.png]

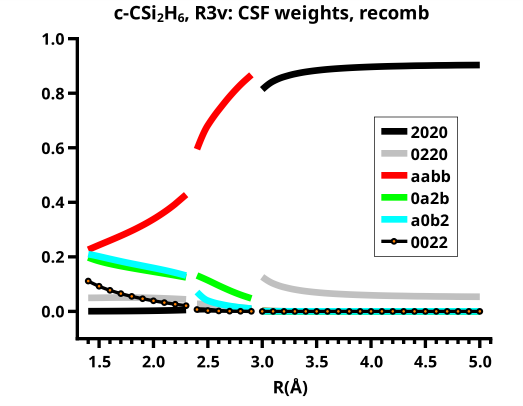

Supplement: Supplementary file 1 [file molecules-30-00612-s001.zip › Figure58.png]

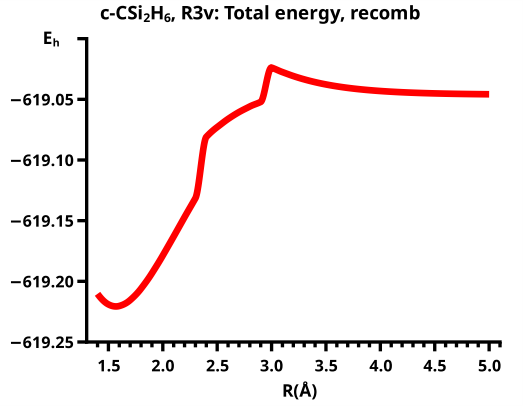

Supplement: Supplementary file 1 [file molecules-30-00612-s001.zip › Figure59.png]

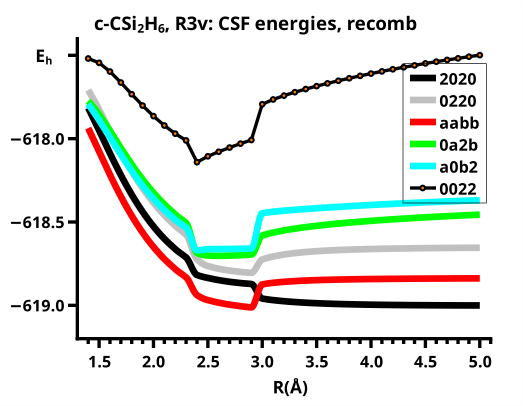

Supplement: Supplementary file 1 [file molecules-30-00612-s001.zip › Figure60.png]

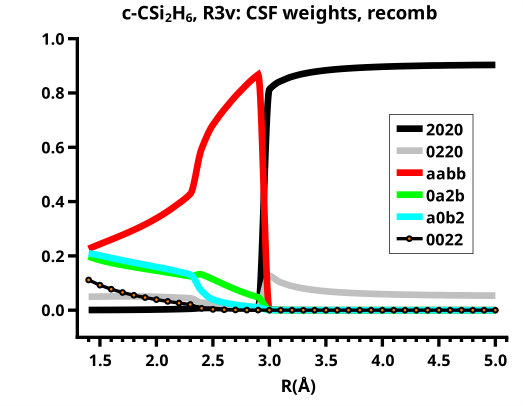

Supplement: Supplementary file 1 [file molecules-30-00612-s001.zip › Figure61.png]

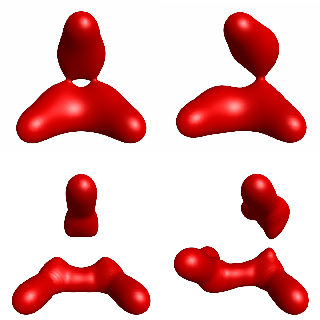

Supplement: Supplementary file 1 [file molecules-30-00612-s001.zip › Figure62.png]

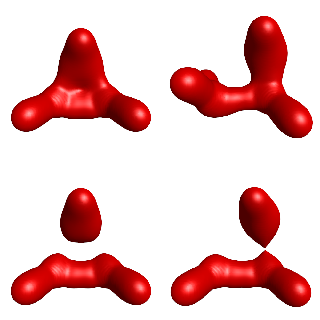

Supplement: Supplementary file 1 [file molecules-30-00612-s001.zip › Figure63.png]

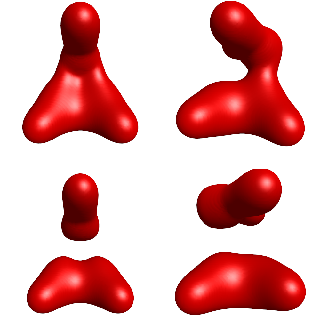

Supplement: Supplementary file 1 [file molecules-30-00612-s001.zip › Figure64.png]
